# Effects of laboratory salmon louse infection on Arctic char osmoregulation, growth and survival

**DOI:** 10.1093/conphys/coz072

**Published:** 2019-11-07

**Authors:** P G Fjelldal, T J Hansen, Ø Karlsen, D W Wright

**Affiliations:** 1 Institute of Marine Research (IMR), Matre Aquaculture Research Station, 5984 Matredal, Norway; 2 Institute of Marine Research (IMR), PO Box 1870, Nordnes, 5817 Bergen, Norway; 3 Department of Primary Industries, Narrandera Fisheries Centre, PO Box 182, Narrandera, New South Wales, Australia

**Keywords:** Arctic char, osmoregulation, physiology, risk index, salmon lice, smolt

## Abstract

High salmon lice (*Lepeophtheirus salmonis*) infestation levels resulting from intensive salmonid sea-cage aquaculture can threaten populations of wild salmonid hosts. This includes anadromous Arctic char (*Salvelinus alpinus*), which rely on short migrations into more productive seawater environments to build energy stores for maturation, spawning and over-wintering in freshwater. Elevated salmon lice burdens may limit the benefits of migration by constraining osmoregulation, growth, survival and reproduction. To test for these effects, we simulated anadromous migration in tanks by transferring individually tagged Arctic char smolts (*n* = 352, averaging 133 g) to seawater where they were infected with salmon lice or left as uninfected controls for 1 month, and then transferring them back to freshwater for 2 months. After the seawater phase, infected post-smolts had a mean of 0.33 (range of 0.09–0.91) mobile lice g^−1^ fish weight. At this point, specific growth rates (SGRs) dropped in infected compared to control fish (0.1% vs. 1.6% day^−1^). Higher plasma Na^+^ and osmolality in infected fish also indicate osmoregulatory impairment. Throughout the study, mortality was 18.2% and 1.7% in infected and control groups, but sexual maturation was low and comparable between groups. Infection intensity correlated positively with mortality rate and plasma Cl^−^, and correlated negatively with SGR and condition factor (CF). CF dropped (ΔCF < 0) at intensities of >0.09 lice g^−1^ fish weight, and intensities of >0.3 causing zero or negative SGRs and increased mortality were particularly concerning. If infection intensities reach these levels in the wild, char could be impacted by growth restrictions and increased mortality rates, which potentially cause shorter migration durations, lowered reproductive success and possibly also selection against anadromy. This study provides vital information for conservation practitioners wanting to understand the physiologically derived burden salmon lice can have on Arctic char populations, and can be used to define thresholds in the monitoring and conservation of Arctic char populations affected by aquaculture-driven salmon lice infestations.

## Introduction

In Norwegian coastal and fjord ecosystems, parasitic salmon lice (*Lepeophtheirus salmonis*) populations that burgeon on commercially produced salmonid hosts in open sea-cages significantly threaten wild salmonids (Taranger *et al.*, 2015; Forseth *et al.*, 2017). By country, Norway is the top producer of sea-caged salmonids at >1.3 million tonnes and 49 billion NOK (http://www.fao.org/fishery/statistics/global-aquaculture-production). Here, salmon lice infestations, treatments and preventive measures result in huge economic losses to the industry, estimated at >5 billion NOK in 2015 (reviewed in Brooker *et al.*, 2018). Despite control efforts, including a nationally enforced threshold of 0.5 adult female salmon lice fish^−1^ in sea-cages (Norwegian Ministry of Fisheries and Coastal Affairs, 2012), the parasite continues to expose wild salmonids to infestation pressures expected to cause mortality (Kristoffersen *et al.*, 2018). Infestation pressures peak in mid-latitudes along the Norwegian coast, where salmon farm density is highest (Kristoffersen *et al.*, 2018) and lice development may be optimized (Samsing *et al.*, 2016; Hamre *et al.*, 2019). However, increasing salmon production at latitudes above 65° N (https://www.fiskeridir.no/fiskeridir/Akvakultur/Statistikk-akvakultur/Akvakulturstatistikk-tidsserier) and elevating coastal water temperatures (Hoegh-Guldberg and Bruno, 2010) will likely exacerbate risks wild salmonids face from salmon lice in northern Norway.

From a conservation viewpoint, aquaculture-driven salmon lice infestations are concerning for the northernmost freshwater fish, Arctic char (*Salvelinus alpinus*), which is a natural salmon lice host and anadromous in its northern distribution area above 65° N (Nordeng, 1983). Arctic char has a specialized life history adapted to living in cold fresh waters. They rely on a short period of 1–2 months in seawater each summer to build an energy surplus for subsequent spawning and over-wintering in freshwater (Johnson, 1980; Jørgensen and Johnsen, 2014). Survival during the winter is linked to marine growth during the previous summer (Jensen *et al.*, 2018). They smoltify at 12–20 cm length and 2–9 years of age, and undertake an average of four seawater runs before reaching sexual maturity, after which they maintain an anadromous life (reviewed in Jørgensen and Johnsen, 2014).

Bjørn and Finstad (2002) studied the abundance and prevalence of sea lice in sea trout (*n* = 72) and Arctic char (*n* = 290) in Finnmark fjord systems with and without salmon seacage aquaculture in the summers of 1992 and 1993, and found no difference in lice abundance between species or size groups, but significantly higher lice levels in the area with seacage aquaculture compared to the area without. Later, Bjørn *et al.* (2001) studied the abundance of sea lice and physiological effects of infection in sea trout (*n* = 95) and Arctic char (*n* = 31) in areas with and without salmon seacage aquaculture in Nordland county in the summer of 1997, and found higher lice levels in the area with aquaculture compared to the area without in both species, and a positive correlation between infection level and plasma Cl in sea trout. Plasma samples were not analysed from Arctic char in that study, where relative abundance of infection (mean number of lice on infected fish g^−1^ fish weight) was 0.5 in the three char that was caught in the area with salmon seacage aquaculture present. The national surveillance program of salmon lice on wild salmonids (NALO) catches wild salmonids (including Arctic char) using traps or nets along the Norwegian coast yearly (method described in Serra-Llinares *et al.*, 2014). Of the 638 wild anadromous Arctic char captured between 2010 and 2018 during the NALO project, 318 have had zero lice, and 320 had infection intensities between 0.001 and 1.49, with a mean infection intensity (II) of 0.12. Of the lice infected char in the survey, 12.2% had infection intensities >0.3 (unpublished data).

Arctic char may suffer a range of sublethal and lethal effects from salmon lice infections. Salmon lice feed on mucus and skin and muscle of fish hosts causing skin lesions and secondary infections that can become life-threatening (Costello, 2006; Thorstad *et al.*, 2015). Infections cause osmoregulatory distress, higher stress levels, anaemia, loss of appetite and reduced growth, lower immunological function, diminished reproductive output and potential death (Costello, 2006; Tveiten *et al.*, 2010; Thorstad *et al.*, 2015). In Arctic char specifically, increased stress and reduced osmoregulatory ability, growth, reproductive investment and survival have been noted for sexually mature adult 5+ year olds infected by salmon lice (Tveiten *et al.*, 2010). However, effects on smaller and immature Arctic char post-smolts remain untested.

The salmon lice risk index is a classification system estimating rates of mortality or premature return to freshwater in wild salmonids based on lice II (Taranger *et al.* 2015). The sum of all indexes gives an estimate of expected reduction in population. Thresholds within the index have been conservatively inferred from previous studies documenting sublethal physiological consequences, in addition to mortality, from salmon lice infections (Taranger *et al.* 2015). The index parameterizes a national-scale model used to quantify the risk of lice-induced mortality in wild Atlantic salmon and regulate the Norwegian salmon farming industry (Kristoffersen *et al.* 2018), based on lice-related mortality predicts of small (<150 g, 100% for >0.3 lice g^−1^, 50% for 0.2–0.3, 20% for 0.1–0.2 and 0% for < 0.1) and large salmonids (>150 g, 100% for >0.15 lice g^−1^, 75% for 0.10–0.15, 50% for 0.05–0.10 and 20% for 0.025–0.05) (Taranger *et al.*, 2015). Currently, a surveillance program is in place to monitor salmon lice infections in sea trout as a proxy for where infection pressures are likely to fall within these categories on Atlantic salmon in a specific region. Despite the development and application of the index across multiple salmonid host species, it does not resolve variations in how salmon lice affect them, and there is a pressing need to verify the appropriateness of salmon lice risk index thresholds for post-smolt Arctic char.

Here, we experimentally examined the effect of salmon lice infection on Arctic char post-smolts during a simulated anadromous migration. Duplicate groups of individually tagged Arctic char yearling (1+) smolts (*n* = 352, averaging 133 g) were gradually transferred from freshwater to seawater and either infected with salmon lice copepodids or kept as uninfected controls in tanks. We aimed to produce infection intensities covering those previously found on wild Arctic char near salmonid farming, and smolt size was similar as in nature (13.9–26.6 cm, average 21.5 cm). Four weeks after their initial freshwater to seawater transfer, a typical seawater phase length for this species, fishes were returned from seawater to freshwater and kept for another 3 months. Response parameters monitored during the experiment were mortality, growth, condition factor (CF), plasma osmolality, plasma K^+^, Na^+^ and Cl^−^ and sexual maturation.

## Materials and methods

The Arctic char (*S. alpinus*) used in the present experiment was of the anadromous Hammerfest strain (Rikardsen *et al.*, 1997). Their parents were brought to IMR’s research station in Matre, Western Norway, as eyed eggs. Fertilization was on 19 October 2016, and first-feeding on 21 February 2017. The water temperature was 6°C during egg incubation, 12°C from first feeding until summer solstice 2017, and natural thereafter. The photoperiod was continuous light from first-feeding until 01 October 2017, simulated natural (Western Norway, 60° N, 5° E) from 01 October 2017 until 04 April 2018, followed by continuous light from 04 April. The fish were PIT-tagged (Glass tag 2, 12 mm, TrackID AS, Stavanger, Norway) on 20 April 2018.

### Ethical statement

All experiments were performed at the Institute of Marine Research, Matre Research Station (60° N, 5° E, Western Norway), which is authorized for animal experimentation (Norwegian Food Safety Authority, facility 110), in accordance with International guidelines certified using Norwegian research permit number 14982.

### Timing of smoltification and seawater transfer

In nature, the char strain we used migrates downstream from early May throughout June (Rikardsen *et al.*, 1997). We used this same time of year for seawater transfer, and employed continuous light to synchronize smoltification. We predicted the optimal timing of smoltification and seawater transfer using a protocol from Jørgensen *et al.* (2007) to calculate how many degree days of continuous light during the spring was required to achieve hypoosmotic ability and elevated gill ATPase enzyme activity. Based on the environmental and physiological data presented by Jørgensen *et al.* (2007) anadromous Arctic char from Finnmark had hypoosmotic ability from 111 up to 260 degree days after the fish were subjected to continuous light, and gill ATPase enzyme activity peaked after 187 degree days. With this knowledge and historic seasonal temperatures at our research facility, we estimated onset of continuous light on 04 April and transfer to seawater on 08 May would optimize the ability of Arctic char to cope with the salinity change. A gradual increase in salinity up to 34 ppt over a 4-day period was selected in order to avoid the combined handling, osmotic and infection stresses. Full salinity was achieved on 12 May. The degree days from onset of continuous light on 04 April to 08 April (15 ppt) and 12 May (34 ppt) were 131 and 165, respectively.

### Experimental setup

On 07 May 2018, 352 char were sedated (Finquel, 0.1 g L^−1^), measured for fork length and body weight and randomly distributed between four 1 m tanks (*n* = 88 per tank). The salinity of the water was gradually increased from freshwater to full strength seawater (34 ppt) in the period 07 to 12 May. On 14 May, two of the tanks were infected with salmon lice (*L. salmonis*) copepodids, while two tanks were un-infected controls. In all four tanks (two infected, two uninfected) the water level was reduced to 20 cm depth, and water flow was stopped before adding copepodids (10 days post-hatch) to the two infection tanks. Then, in all tanks, the water flow was turned back on after 10 min, with low flow rate for 10 min, followed by normal flow. In total, 17 600 copepodids were used to infect the fish (8800 copepodids per tank), giving an average infection pressure of 100 lice per fish. The challenge was terminated 28 days post-infection (11 June 2018) when a seawater infection sampling took place. Sampling involved netting one fish at a time from their respective tanks, sedation (0.01 g L^−1^, Aquacalm vet., Scan Aqua AS, Årnes, Norway), reading the PIT tag, measuring fork length and body weight and counting lice. Counts of lice per fish included all lice remaining in individual anaesthetic water containers they were place in, in addition to those on live and dead fish. By the time of sampling, mobile preadult II male and preadult I and II female stages had developed at 8.9°C (Hamre *et al.*, 2019). Only lice number and not the stage was quantified. In addition, blood was collected from 15 random fish per tank. Blood was centrifuged and plasma stored at −80°C until analysis.

Lice for the infection were produced from an outbred strain that had been maintained at approximately 9°C at the Institute of Marine Research lice hatchery using methods described in Hamre *et al.* (2009). In the fish tanks, the photoperiod in seawater was continuous light, and the water temperature was 8.9°C. After the sampling the tank water was changed to freshwater, and the photoperiod was shifted to natural light.

A first freshwater sampling occurred on 17 July 2018, when all fish were sedated (Finquel 0.1 g L^−1^), and had their PIT tag, fork length and body weight recorded. During this sampling, the fish in each tank were randomly split in two and allocated between two tanks in order to reduce the stocking density, using a total of eight tanks. The fish were reared in these tanks until 24 September 2018 when the experiment was terminated and a final freshwater sampling was performed once the fish were killed by an overdose with anesthetic (Finquel 0.5 g L^−1^). At this point, PIT tag number, sex and maturity status based on examination of dissected gonads, length and weight were recorded.

**Figure 1:**
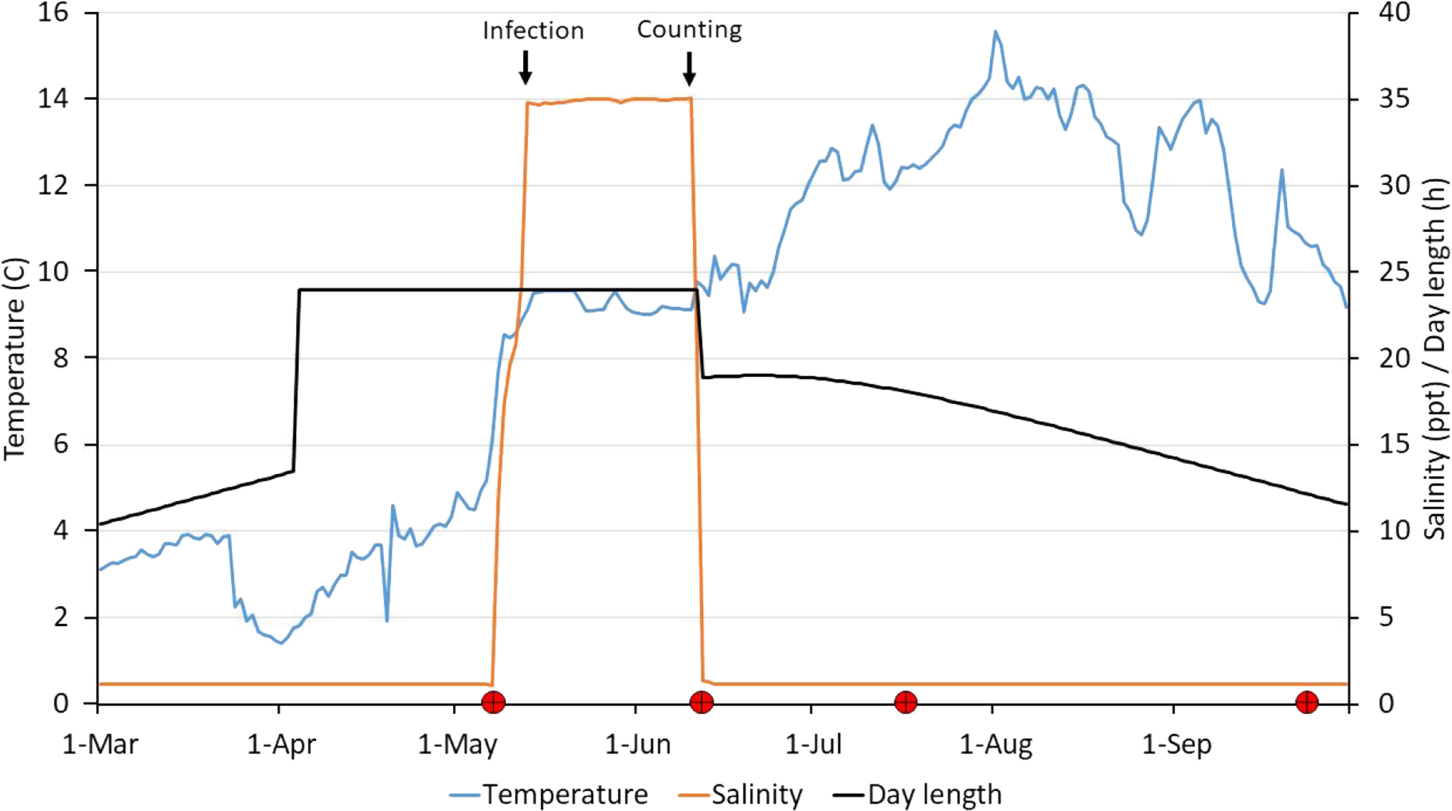
Water temperature and salinity, and photoperiod throughout the experimental period. Black arrows indicate time of salmon lice infection (14 May) and counting (11 June). Red circles indicate sampling points.

The outline of the experiment and environmental data are presented in Fig. 1. No fish were killed during the samplings, and all fish went through the complete experiment (07.5.18–24.09.18) and were measured at each sampling point.

### Plasma analysis

Plasma ion levels (Na, Cl, K) were detected by ion selective electrodes using a 9180 Electrolyte Analyser (Roche Diagnostics, Minnesota, USA). Plasma osmolality was determined by freeze point determination (Fiske microosmometer Model 210, Norwood, Mass, USA). One sample had to low volume for complete analysis. Thus, ions were measured in 59 samples, and osmolality in 60.

### Calculations and statistical analysis

II was calculated using *II* = *Ln Fw*^−1^, where *Ln* was number of lice on infected fish and *Fw* was body weight (g) of infected fish at time of counting lice on 11 June.

The CF was calculated using *CF* = (*WL*^−3^)100, where *W* was the live body weight (g) and *L* was the fork length (cm). Specific growth rate (SGR) was calculated using: SGR = (*e^G^*-1)100, where *G* = (ln(*X_2_*) − ln (*X_1_*))/(*t_2_* − *t_1_*), *X_2_* and *X_1_* were the body weights at times *t_2_* and *t_1_*. Change in CF (ΔCF) was calculated using: Δ*CF* = *CF_2_*—*CF_1_*, where *CF_1_* was CF on sampling number 1, and *CF_2_* was CF on sampling number 2.

The data were analysed using Statistica version 12 (StatSoft, Inc., 2300 East 14th Street, Tulsa, Oklahoma, USA), except for broken line regression analyses in R version 3.3.1 (R Core Team, 2016, R Foundation for Statistical Computing, Vienna, Austria). Results are shown as means with their standard errors. Sampling point differences within different parameters were tested by two-way nested ANOVAs with tank as random factor nested in treatment. Possible significant correlations between II and measured parameters were tested by product-moment and partial correlations. Potential II breakpoints, above which measured parameters became substantially more impaired, were also investigated using the ‘segmented’ package (Muggeo, 2003). Breakpoints were only considered to be present when there was a significant change in slope above and below the breakpoint identified using the Davies test. *P* < 0.05 was classified as statistically different.

## Results

### II

Mean II (lice g^−1^) in the two infected tanks were 0.29 (± 0.013) and 0.37 (± 0.017), and 0.33 (± 0.011) overall (Supplementary Fig. 1). This equated to mean numbers of lice fish^−1^ of 42 (± 1.7) and 47 (± 2.0) in the two tanks, and 44 (± 1.3) across all individuals, with 100% prevalence. No lice were present after 4 weeks in freshwater on 17 July.

### Mortality

In total, 32 (18.2%) and 3 (1.7%) individuals died during the experimental period in the lice infected and control groups, respectively. In the control group, one fish died on transfer to experimental tanks on 07 May 2018, and handling stress was likely the cause of death. The two remaining control fishes died mid-September, more than 10 weeks after the salinity had been changed from seawater to freshwater. In the infected group, the mortalities occurred between 04 and 17 June, pre-dominantly towards the end of the seawater period that ended on 11 June. Of the 32 deaths, 26 were in seawater, and 6 in freshwater.

### Osmoregulation

Plasma Na^+^ and osmolality was significantly higher, while plasma K^+^ significantly lower (two-way nested ANOVA, *P* < 0.05) in lice infected than in control fish on 11 June, 4 weeks post-infection in seawater (Table 1).

**Table 1:** Plasma Na, Cl, K and osmolality in Arctic char (*Salvelinus alpinus*) on 11 June in seawater, 4 weeks post-infection with salmon lice (*Lepeophtheirus salmonis*) copepodites. *N* = 29–30 per treatment group (14–15 per duplicate tank). The fishes were infected (mean II 0.33 lice g^−1^) on 14 May, 7 days after change to seawater, and number of lice counted on 11 June

Plasma parameter	Salmon lice infected	Control	*P*-value^*^
Na (mmol L^−1^)	193.9 ± 4.0	165.6 ± 0.8	***0.0414***
Cl (mmol L^−1^)	164.9 ± 5.6	132.4 ± 0.7	0.2356
K (mmol L^−1^)	2.9 ± 0.1	3.4 ± 0.1	***0.0026***
Osmolality (mOsm kg^−1^)	399.4 ± 8.3	349.8 ± 3.9	***0.0353***

^*^Number in italic and bold indicates a significant difference (two-way nested ANOVA, *P* < 0.05).

**Figure 2:**
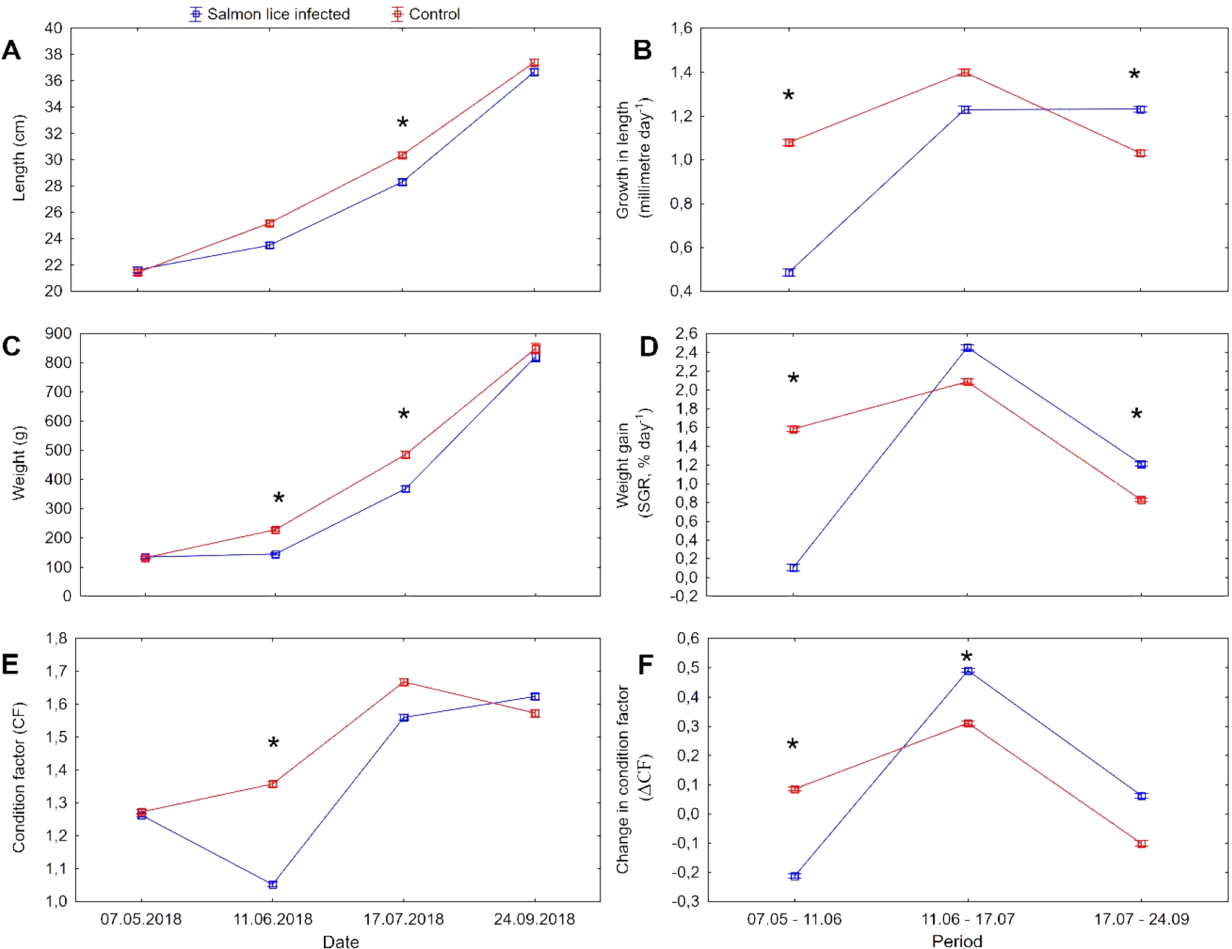
Growth parameters (mean ± SE) in anadromous Arctic char (*Salvelinus alpinus*) infected with salmon lice copepodids (*Lepeophtheirus salmonis*, II, 0.33 lice g^−1^) on 14 May 2018, and counted for lice and shifted from sea- to freshwater on 11 June 2018. Un-infected fish served as control. Length (**A**), growth in length (**B**), weight (**C**), weight gain (**D**), CF (**E**), change in CF (**F**). *N* = 176 fish per group; 88 per tank. ‘^*^’ indicates significant difference (nested ANOVA, *P* < 0.05) within sampling point.

**Figure 3:**
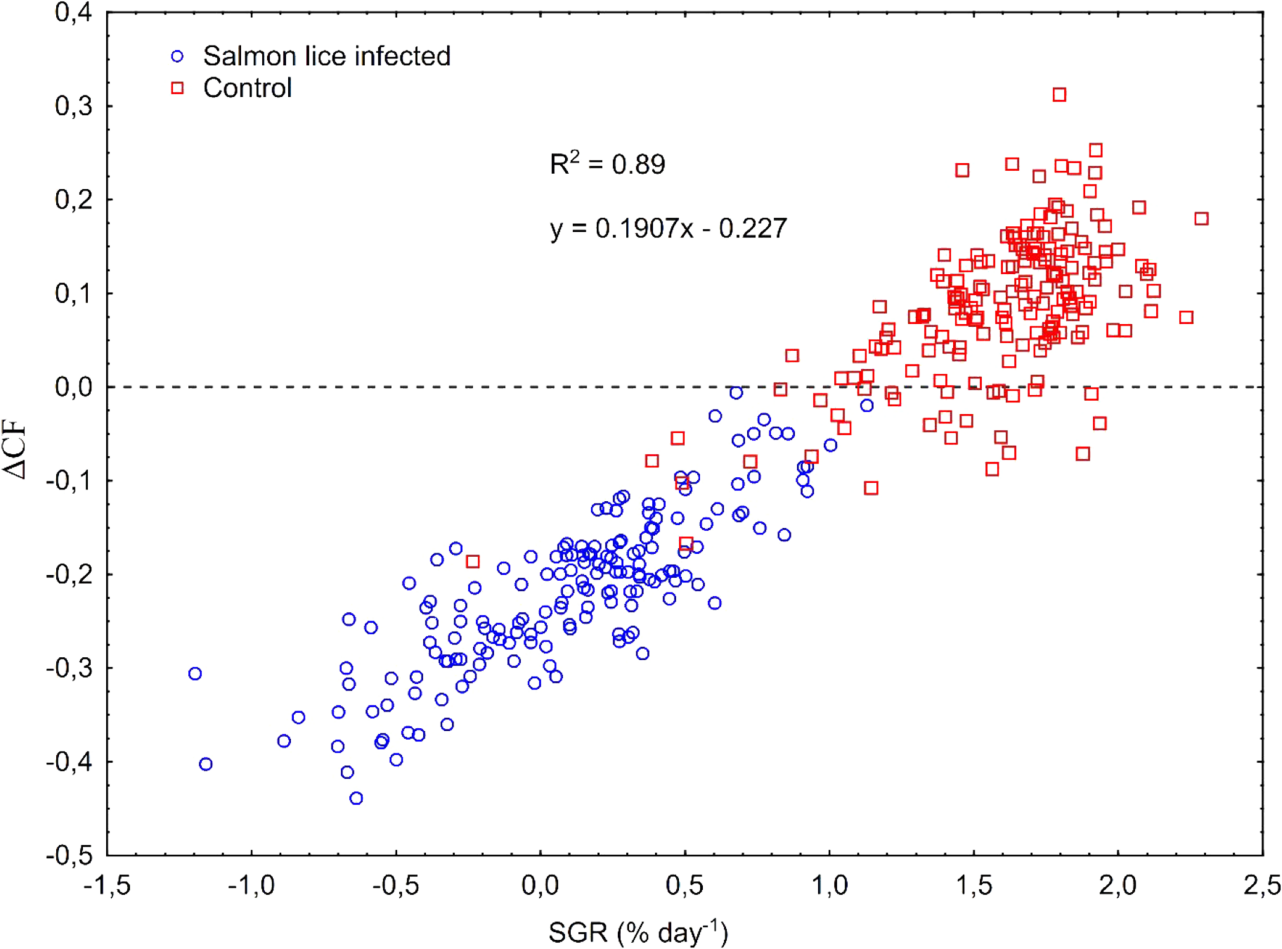
Correlation between SGR (% day^−1^, 07 May to 11 June) and change in CF (ΔCF), calculated for the period 07 May to 11 June, in anadromous Arctic char (*Salvelinus alpinus*) infected with salmon lice (*Lepeophtheirus salmonis*) copepodids. The fishes were infected on 14 May (mean II, 0.33 lice g^−1^), and number of lice counted on 11 June.

### Growth and sexual maturation

No differences (two-way nested ANOVA, *P* > 0.6) in length, weight and CF were present between the treatment groups at the start of the experiment on 07 May (Fig. 2A−F). On 11 June, 4 weeks post-infection in seawater, lice infected fish had significantly lower weight and CF compared to control fish. The calculated mm day^−1^, SGR and ΔCF in the period between 07 May and 11 June were significantly lower in the lice infected compared to the control group. There was an overall significant positive correlation between SGR and ΔCF for the period 07 May to 11 June, and SGR > 1.18 was required to avoid a drop in CF (ΔCF > 0) (Fig. 3). All infected fish had ΔCF < 0, while 83% of the control fish had ΔCF > 0 (Fig. 3). On 17 July, 4 weeks after transfer from seawater to freshwater, the lice infected group had significantly lower length and weight compared to the control group. The calculated ΔCF in the period between 11 June and 17 July was significantly higher in the infected compared to the control group. On 24 September, there were no treatment effects on absolute values in size or CF, but both SGR and mm day^−1^ were significantly higher in the infected compared to the control group between 17 July and 24 September. The incidences of maturation were 11.7% and 4.5% in infected males and females (8.3% total maturation), and 6.2% and 7.7% in control males and females (6.9% total maturation).

### II thresholds

**Figure 4:**
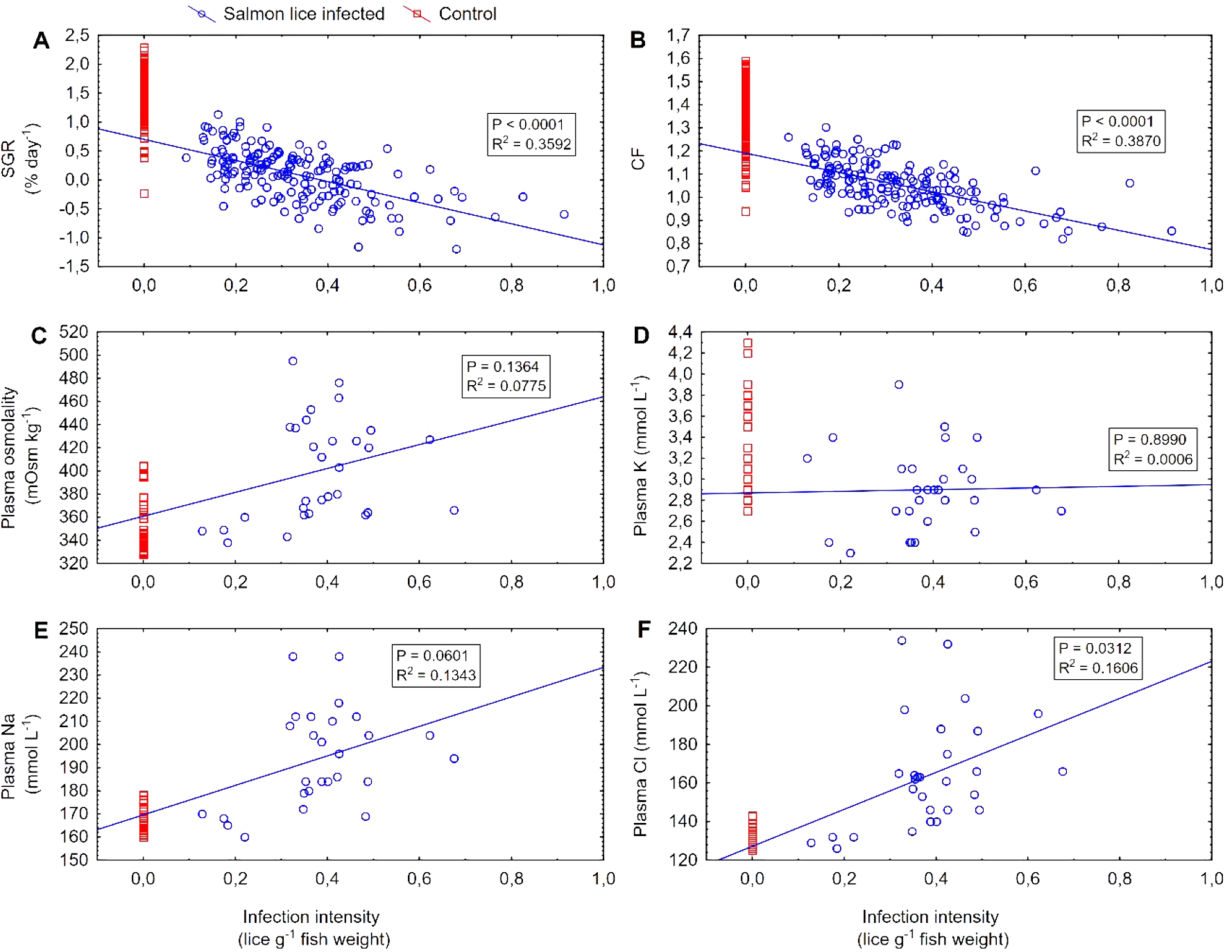
Correlations between SGR (% day^−1^, 07 May to 11 June) (**A**), CF (11 June) (**B**), plasma osmolality (mOsm kg^−1^) (**C**), plasma K (mmol L^−1^, 11 June) (**D**), plasma Na (mmol L^−1^, 11 June) (**E**), plasma Cl (mmol L^−1^, 11 June) (**F**) and II (lice g^−1^, June 11) in anadromous Arctic char (*Salvelinus alpinus*) infected with salmon lice (*Lepeophtheirus salmonis*) copepodids. The fishes were infected on 14 May (mean II 0.33 lice g^−1^), and number of lice counted on 11 June. Trend lines, *P* and *R*^2^ values are based on data from the salmon lice infected group. Data from the un-infected control group are included for comparison.

For infected individuals, more mortalities were recorded at higher IIs (Table 2), with no mortalities at 0.09 (lowest level recorded) − 0.2 lice g^−1^ and all fish dying at ≥0.7. II was significantly correlated with SGR, CF and plasma Cl^−^, but not plasma K^+^ or Na^+^ (Fig. 4A−F). Plasma Cl^−^ rose with higher IIs, and were particularly elevated at ≥0.3 lice g^−1^ (Table 2). There was a decrease in both SGR and CF with increasing II, and 0.3–0.4 lice g^−1^ gave zero growth, while ≥0.4 gave negative SGRs (Table 2). ΔCF was the most sensitive response parameter, with negative values for all infection levels and decreasing values with increasing II (Table 2). No breakpoints were identified in relationships between II relative to SGR, CF or blood parameters (Davies tests, *P* ≥ 0.1).

## Discussion

### Salmon lice effects on Arctic char post-smolts

Salmon lice infection had significant physiological consequences for Arctic char post-smolts during a simulated 4-week anadromous migration. Mobile preadults at 0.33 lice g^−1^ 28 days post-infection impaired osmoregulation, ceased SGR and reduced CF. In addition, substantial mortalities occurred. After an extended subsequent period in freshwater, previously infected individuals shed all lice, displayed compensatory growth and had normal incidences of sexual maturation.

Mortalities in infected fish were found during and soon after the seawater phase. Initial mortalities at 21 days post-infection coincided with the estimated development of preadult I male lice at 20 days at 8.9°C (Hamre *et al.*, 2019). Previous studies also link preadult development to the onset of deaths in experimental infection challenges (Grimnes and Jakobsen, 1996; Bjørn and Finstad, 1998). Extending the seawater phase would have undoubtedly increased infected fish mortalities (Grimnes and Jakobsen, 1996; Bjørn and Finstad, 1998). The cessation of mortalities soon after transfer back to freshwater provided further evidence that premature freshwater returns circumvent the short-term effects of salmon lice infection (Wells *et al.*, 2007).

The present char smolts were subjected to continuous light to stimulate smoltification (Jørgensen *et al.*, 2007), and the transfer to seawater was in the middle of the predicted smolt window for anadromous Arctic char (see ‘Materials and Methods’). The char in the present control group had 100% survival and strong appetite throughout the seawater period at 9°C, reflected by a high SGR of 1.6% and a substantial increase in CF, which suggests that the fish were smoltified and preadapted for seawater. Arnesen *et al.* (1995) used the same char strain at salinities between 0 and 35 ppt from 01 April and 30 days onwards at 8°C and reported SGRs from 0.85–1.26%, with no differences between salinities. Wild anadromous char may double their weight during 4–6 weeks in seawater (Jørgensen and Johnsen, 2014). Our control fishes were expected to double their weight in 6.3 weeks if left in seawater for this period, indicating preparedness may have been comparable to those in the wild.

**Table 2:** Mortalities (%), SGRs (% day^−1^, 07 May to 11 June) and CFs (CF, 11 June) in anadromous Arctic char (*Salvelinus alpinus*) categorized according to II (lice g^−1^, 11 June). Category ‘0’ is the uninfected control fish. The infected fishes were infected with salmon lice (*Lepeophtheirus salmonis*) copepodids on 14 May (mean II 0.33 lice g^−1^), and number of lice counted on 11 June

II **(lice g**^**−1**^**)**	**Fish numbers** ^*^	**Mortality (%)**	**SGR** **(% day** ^**−1**^ **)**	**ΔCF**	**CF**	**Blood samples**	**Cl** **(mmol L** ^**−1**^ **)**
0 (control)	178	1.7	1.58	0,08	1.36	30	132
0.09–0.2	35	0.0	0.44	−0.15	1.14	3	129
0.2–0.3	45	2.2	0.29	−0.20	1.08	1	132
0.3–0.4	41	14.6	0.03	−0.23	1.04	12	165
0.4–0.5	30	23.3	−0.17	−0.27	1.00	11	173
0.5–0.6	8 (9)	55.6	−0.34	−0.28	0.94	—	—
0.6–0.7	6 (7)	57.1	−0.42	−0.28	0.92	2	181
≥ 0.7	3 (9)	100.0	−0.51	−0.29	0.93	—	—

^*^Numbers in brackets include fishes that were not recorded for length and weight at time of death, and are only included in the mortality calculation. IIs in those fish were calculated based on start weight on 07 May.

Osmoregulatory dysfunction was evident in infected fish in seawater. Infected individuals exhibited clearly heightened Na^+^ concentrations. While this was not the case for Cl^−^ ions, a positive correlation between Cl^−^ concentration and II suggested more infected individuals were in greater distress. In seawater, teleosts actively excrete Na^+^ and Cl^−^ to maintain ionic balance (Marshall, 2002), and elevated plasma Na^+^ (Clarke, 1982) and Cl^−^ (Jørgensen *et al.*, 2007) are indicators of impaired hypoosmoregulation. The development of lice past sessile stages, enabled here, is known to trigger greater osmoregulatory imbalance in fish, as feeding and skin damage intensifies (Grimnes and Jakobsen, 1996; Bjørn and Finstad, 1998; Wells *et al.*, 2006). Increased plasma cortisol, widely reported during similar lice infection challenges in salmonids (Grimnes and Jakobsen, 1996; Wells *et al.*, 2006; Wells *et al.*, 2007; Tveiten *et al.*, 2010), is involved in stress responses that elevate epithelia membrane permeability and also explains greater ion uptake (Bonga, 1997).

Plasma potassium was unexpectedly lower in infected fish. Potassium content is higher in seawater than fish plasma (Partridge and Lymbery, 2008), and excretion occurs via specific potassium channels in the gills (Furukawa, *et al.*, 2012). It is a key electrolyte in osmoregulation with its role in the branchial sodium–potassium pump (Na^+^/K^+^-ATPase; NKA). NKA is localized in the basolateral membrane of mitochondrial rich chloride cells in the gills (Marshall, 2002), where cortisol impacts its activity (McCormick *et al.*, 2008). In seawater, potassium mostly recycles over the basolateral membrane of chloride cells to support the NKA pump, while a smaller amount of potassium is excreted over their apical membrane (Marshall, 2002). Atlantic salmon postsmolts have shown to elevate gill NKA enzyme activity in response to infection with salmon lice (Nolan *et al.*, 1999), and Farrell (2011) reported chloride cell proliferation in the gills of salmon lice infected Atlantic salmon. The potential physiological effects of low plasma potassium include hypokalemia, which induces arrhythmias and heart failure in humans (Skogestad and Aronsen, 2018).

SGR and ΔCF were significantly reduced in infected fish while in seawater. Stress, dehydration, lowered feeding activity typically induced by preadults but also earlier stages (see Wells *et al.*, 2006) and other infection-associated responses such as increased jumping behaviour may have elevated energy consumption and lowered feed uptake to affect growth and condition of infected fish (reviewed by Thorstad *et al.*, 2015). Compensatory growth and restored condition of infected fish after returning to freshwater and removing their lice highlighted the effectiveness of river re-entry as a strategy to physiologically cope with salmon lice infection (Wells *et al.*, 2007). Naturally, in freshwater, salmon lice copepodid infections are avoided (Heuch, 1995; Bricknell *et al.*, 2006), and later host-attached stages perish after several weeks (Finstad *et al.*, 1995).

### Ecological relevance

We simulated a 4-week anadromous migration in tanks to study salmon lice effects on Arctic char post-smolts. Infection intensities, averaging 0.33 lice g^−1^ and ranging from 0.09–0.91, covered those known in wild Arctic char (NALO project, unpublished data). The infection was also delivered in a single pulse of copepodids soon after seawater acclimation, thought to reflect natural infection dynamics, particularly in waters affected by salmonid farming (Wells *et al.*, 2006). The size, age and smoltification status of the fish also closely matched that of anadromous Arctic char performing their first seawater migration (Jørgensen and Johnsen, 2014). Furthermore, the 1-month stay in seawater was typical of wild Arctic char (Jørgensen and Johnsen, 2014). Although ecologically relevant in these respects, several factors would differ in nature relative to our controlled tank experiment.

Firstly, food resource availability varies in nature. Here, nutritious commercial diets were oversupplied in freshwater and seawater. However, food availability is drastically lower in natural freshwater compared to marine habitats in the temperate latitudes Arctic char reside in (Gross *et al.*, 1988). If food availability in freshwater was lowered in our experiment to mirror the experience of wild Arctic char, the smaller sizes and worse condition of infected fish in seawater may have persisted through the following freshwater period. In addition, salmon lice infection effects on sexual maturation may have been detected. A study finding salmon lice effects on reproductive development and output in adult Arctic char did not feed fish in freshwater periods before and after the seawater phase to simulate natural conditions (Tveiten *et al.*, 2010).

Secondly, wild fish compromised by salmon lice could suffer heightened levels of indirect mortality from predation, competition for prey, poor environmental conditions and other immunological challenges (Thorstad *et al.*, 2015) that could not be measured in our experiment.

Thirdly, returning to freshwater is a possible behavioural response available to migrating wild Arctic char suffering salmon lice infection that was prevented in our study. Wild salmonids, particularly sea trout, infected by salmon lice are known to bring forward their freshwater re-entry time to reinstate normal osmoregulatory function and survive (Birkeland, 1996; Birkeland and Jakobsen, 1997; Wells *et al.*, 2007). Therefore, wild Arctic char in seawater experiencing the same infection intensities to the current study may have returned to freshwater early, before osmoregulation, growth and survival were substantially affected. While avoiding these short-term consequences, cutting short summer migrations to gather energy stores could incur an overall energy budget cost that does not support spawning, over-wintering and long-term survival in Arctic char (Jensen *et al.*, 2018). To avoid a drop in CF in seawater (ΔCF < 0), the present char post-smolts needed SGR’s above 1.18, showing how specialized they are for short seawater growth spurts. Anadromous and permanently freshwater resident Arctic char populations coexist within the same gene pool (Nordeng, 1983). Thus, increased salmon lice infestations potentially risk selection against anadromy.

Fourthly, the present lice II was possibly higher and more intense than what is experienced in the wild. Also, in experimental lice infection studies, it is impossible to know which fish gets infected and by how many lice, nor how many lice are retained to motile stages. The large variation in II in the current study may reflect that some fish are better in ridding themselves of lice than others. However, repeated sedation and handling of fish and lice would create an un-natural stress situation for both host and parasite. The number of char available for the present experiment limited the experimental design and did not allow us to study which fish gets infected by how many lice, and how many lice were retained to maturity. Including a lower II level and a more refined experimental design, including more fish and rearing units, would have made a more eco-relevant study. For refinement of laboratory methods and reporting of salmon lice infection trials, appropriate sample size, replication and availability of sufficient copepodids are suggested as the most critical factors (reviewed in Wagner *et al.*, 2008). These factors were all considered in the present study.

### Salmon lice risk index implications

Our study provides new physiological information on how salmon lice II affects Arctic char post-smolts. Although the present experimental design has shortcomings with regard to ecological relevance, some possible implications of the obtained data on the salmon lice risk index—used to model wild salmonid mortality from aquaculture-driven increases in salmon lice—are addressed in this section.

It is important to note that infection intensities may have varied due to lice losses during our experiment. Only 44% of the lice used for our infection challenge were counted as preadults, similar to another study where only 58% of lice used at infection were found as preadults in larger Arctic char (Tveiten *et al.* 2010). Initial infection success along with subsequent mortality and dislodgement will decrease salmon lice numbers relative to those encountered during laboratory experiments (Wagner *et al.*, 2008; Bui *et al.* 2017). For Atlantic salmon, salmon lice retention after infection success can be high at 96% (Bui *et al.* 2017), and modelling predicts 100% of the lice infecting Atlantic salmon survive until preadult and adult stages (Kristoffersen *et al.* 2018). Post-infection retention rates are untested in Arctic char, but can be much lower in other salmonid host species at 38–84% (Bui *et al.* 2017), and need to be addressed by models.

The salmon lice risk index is partly formulated using direct mortality rates from salmon lice infection (Taranger *et al.* 2015). Atlantic salmon and sea trout post-smolts are reported to die from infection intensities of 0.75 and 1.0 lice g^−1^, respectively, before lice reach the adult stage (Bjørn and Finstad, 1998; Grimnes and Jakobsen, 1996). We observed nine Arctic char post-smolts harbouring ≥0.7 lice g^−1^ dying while preadults were present, suggesting an equivalent direct mortality threshold exists for this species.

The index also accounts for sublethal effects of salmon lice infection on stress, osmoregulation and reproductive output (Taranger *et al.* 2015). For instance, thresholds for small sea trout are partially based on 0.35 lice g^−1^ abruptly altering osmoregulatory, metabolic and stress measures in sea trout post-smolts (Wells *et al.* 2006). We found a similar pattern for osmoregulatory parameters in infected Arctic char post-smolts, even though no clear II thresholds were identified. Cl concentrations increased from 129–132 to 165–181 within II categories below and above 0.3 lice g^−1^ (Table 1).

SGR and ΔCF are other important sublethal effects to consider for Arctic char in the salmon lice risk index. Winter survival is reliant on growth during the previous summer in Arctic char migrants (Jensen *et al.*, 2018), so any reductions in growth or condition owing to salmon lice could ultimately cause mortality. Overall, we found SGR in infected post-smolts as low as 0.1% day^−1^ relative to controls at 1.6% day^−1^ from an II of 0.33 lice g^−1^. Our results align well with the stable SGR found in sea trout post-smolts at the end of an infection with ≥0.42 lice g^−1^ (Bjørn and Finstad, 1998). However, we also observed SGRs at 0.44% day^−1^ within an II category of 0.09–0.2 lice g^−1^, zero or negative SGRs with infection intensities > 0.3, and negative ΔCF were found at all II categories. Comparative studies on salmon lice effects between salmonid host species (e.g. Bui *et al.* 2017) and fish sizes, nuanced to capture differences in their ecology, will ensure the salmon lice risk index is optimally developed to conserve and manage all wild salmonid stocks alongside salmonid farming.

## Supplementary Material

supplementary_figure_coz072
