# Supplementary figures and images for: Effects of laboratory salmon louse infection on Arctic char osmoregulation, growth and survival
Source: Conserv Physiol. 2019 Nov 7;7(1):coz072. doi: 10.1093/conphys/coz072 (PMC6839430; doi:10.1093/conphys/coz072)

**Supplementary Figure 1**


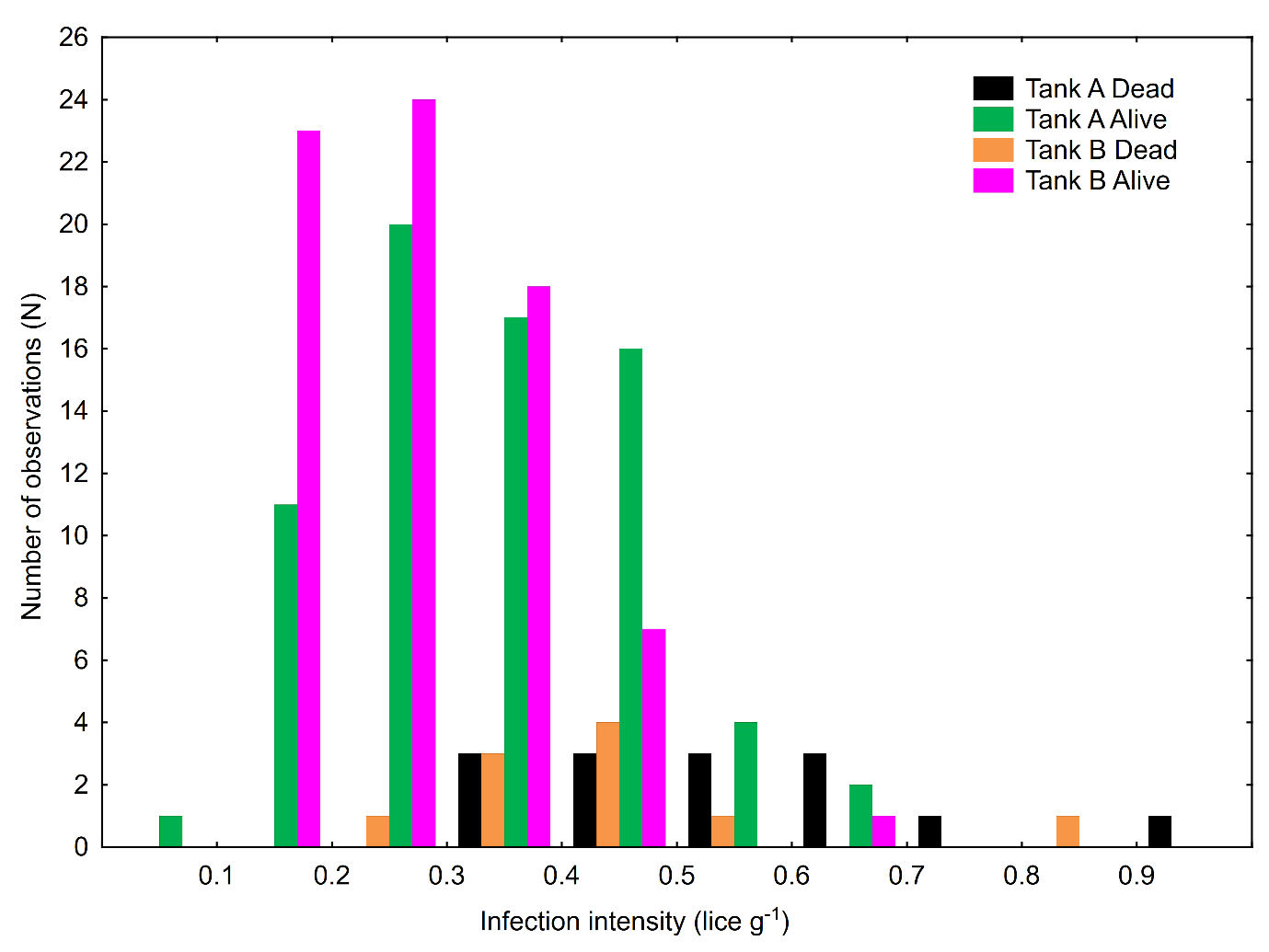

Supplement: supplementary_figure_coz072 [file supplementary_figure_coz072.docx]
